# *CD44*, *IL-33*, and *ST2* Gene Polymorphisms on Hepatocellular Carcinoma Susceptibility in the Chinese Population

**DOI:** 10.1155/2020/2918517

**Published:** 2020-09-29

**Authors:** Xiaolan Pan, Meiqin Li, Lei Huang, Dan Mo, Yihua Liang, Zhaodong Huang, Bo Zhu, Min Fang

**Affiliations:** ^1^Department of Clinical Laboratory, The Affiliated Tumor Hospital of Guangxi Medical University, Nanning, 530021 Guangxi, China; ^2^Department of Surgery, Maternal and Child Health Hospital of the Guangxi Zhuang Autonomous Region, Nanning, China

## Abstract

The interleukin- (IL-) 33/*ST2* axis plays a pivotal role in tumorigenesis through influencing cancer stemness and other mechanisms. *CD44* is one of the critical markers of hepatocellular carcinoma (HCC) among the cancer stem cells (CSCs). There is still a lack of *CD44* gene single-nucleotide polymorphisms (SNPs) combined with *IL-33*/*ST2* pathway single-nucleotide polymorphisms in HCC susceptibility analysis literature, although *CD44* and *IL-33*/*ST2* have been reported separately in human cancers. This study is aimed at investigating the relationship between *CD44*, *IL-33*, and *ST2* SNPs and HCC susceptibility and clinicopathological features. We analyzed 565 HCC patients and 561 healthy controls in the Chinese population. The genes for *CD44*rs187115A>G, *IL-33* rs1929992A>G, and *ST2* rs3821204G>C were typed using the SNaPshot method. We found that the distribution frequencies of *CD44* and *ST2* alleles and genotypes in both the HCC case group and the control group were statistically significant (*p* < 0.05). The results showed that individuals carrying at least one G allele of the *CD44* rs187115 gene were at a higher risk than the AA genotype carriers (*p* = 0.007, odds ratio (OR) = 1.429, 95% confidence interval (CI): 1.102–1.854). Similarly, individuals with at least one C allele of *ST2* rs3821204 had a higher risk of HCC than those with GG genes (*p* ≤ 0.001, OR = 1.647, 95% CI: 1.296-2.093). Combining the haplotype analysis of the 3 loci suggested that *CD44* rs187115, *IL-33* rs1929992, and *ST2* rs3821204 are associated with the risk of HCC and could potentially serve as useful genetic markers for HCC in some populations of China.

## 1. Introduction

Hepatocellular carcinoma (HCC) is a leading cause of cancer-related death worldwide. The annual global incidence of primary liver cancer is 841,000; the death rate is 782,000; and it is the second highest mortality rate among males [1]. The mortality rate of liver cancer in China is the second highest among malignant tumors (new liver cancer accounts for more than 50% of the world's total) and is following a trend of annual increase [2]. Surgical resection, liver transplantation, and tumor ablation are the potential curative therapies; however, these treatment options are only applicable to patients in the early stage of disease [3, 4]. Due to the highly malignant potential of HCC, diagnosis is not usually made until an advanced stage, at which point there are no effective therapies to be offered [5]. Therefore, it is of pressing importance to identify which genes are responsible for susceptibility to HCC. Single-nucleotide polymorphism (SNP), a well-defined molecular biomarker, has been widely applied in HCC susceptibility evaluation [6, 7].

Various factors, including viral infection and cirrhosis, may be involved in the development of HCC [8, 9]. Tumor stem cells (CSCs), a small number of stem cell-like cancer cell subsets in tumor tissues, are characterized by both cancer cells and stem cells and are decisive in the initiation of HCC formation, growth, and metastasis [10, 11]. The CSC marker *CD44* is essential in several malignancies, including HCC, and is the primary adhesion molecule of the extracellular matrix, playing a pivotal role in tumor cell differentiation, invasion, and metastasis [12, 13]. Recent studies have suggested that in the development of HCC, *CD44*^+^ cells were entwined with the genetic processes involved in cancer invasion and metastasis [14, 15]. HCC was often detected following the onset of cirrhosis, and the majority of patients worldwide with HCC have underlying cirrhosis [16]. The *IL-33* gene, located on chromosome 9 (9p24.1), binds to *ST2* and forms a trimer with IL-1R accessory protein (IL-1RAcP), which recruits downstream signaling molecules through the Toll/IL-1R (TIR) domains of *ST2*. The signaling pathways of nuclear factor kappaB (NF-*κ*B), activator protein 1 (AP-1), and mitogen-activated protein kinase (MAPK) are then activated, and the subsequent upregulation of the gene expression of proinflammatory cytokines leads to hepatic fibrosis [17–19]. Although *CD44* and *IL-33*/*ST2* have been well documented in human cancer metastasis or prognosis, respectively, evidence of the *CD44* gene SNPs combined with *IL-33*/*ST2* pathway functional polymorphisms in HCC susceptibility and clinical characteristics is scarce. We aimed to investigate in this study the association of these 3 polymorphisms with demographics, etiology, clinical features, and susceptibility to HCC.

## 2. Materials and Methods

### 2.1. Subjects and Clinical Data

The participant cohort was continuously recruited from September 2016 to December 2018 at the Affiliated Tumor Hospital of Guangxi Medical University. All patients were newly diagnosed and pathologically confirmed as having HCC, according to the American Association for the Study of Liver Diseases guidelines [20]. Ultimately, the study included 565 participants in the case group, 487 males and 78 females, aged 10–89 years. The control group was matched for sex and age and included a total of 561 cases (male = 488, female = 73), aged 22–78 years. The healthy control group patients were free of hypertension, diabetes, dyslipidemia, liver diseases, and cancer. Demographic data included medical record number, gender, age, drinking history, smoking history, tumor stage, and related biochemical indicators. All participants signed a written consent form after being informed of the study details. The Judging Committee approved the study of the Affiliated Tumor Hospital of Guangxi Medical University.

### 2.2. DNA Extraction and Genotyping Assays

We collected 2 ml of fasting peripheral blood from each participant and placed it in ethylenediaminetetraacetic acid- (EDTA-) K2 anticoagulant tube. After thorough mixing, it was stored in a refrigerator at -80°C for DNA extraction from the genome. Genomic DNA was extracted from the 2 ml of peripheral blood using a commercial kit according to the manufacturer's instructions (Adelaide, Beijing, China) and stored at –80°C before genotyping. The genotyping of *CD44* rs187115, *IL-33* rs1929992, and *ST2* rs3821204 was performed using the SNaPshot method as previously described [21], and negative controls were used in each test to ensure accuracy of the genotype evaluation. The primers are listed in Table 1.

### 2.3. Statistical Analysis

First, we assessed whether genotype frequencies were in the Hardy-Weinberg equilibrium (HWE). The Pearson two-sided chi-squared (*χ*^2^) test was used to analyze the HWE law to confirm whether the population of the study samples was representative of the populace [22]. When a *p* > 0.05 value was observed, the samples were representative of the population. Then, the *χ*^2^ test was used to examine the difference in clinicopathological features between the case group and the control group. The distribution data of alleles and genotypes were compared using the *χ*^2^ test and logistic regression analysis, and the relative risk was expressed by odd ratios (OR) and their 95% confidence intervals (CI). Additionally, unconditional logistic regression was used to correct the effects of confounding factors such as gender, age, drinking history, and smoking history on OR values and 95% CI. The haploid model analysis of the gene interaction was performed using the SHEsis software (http://analysis.bio-x.cn/myAnalysis.php) [23]. eQTL are regions of the genome containing DNA sequence variants that influence the expression level of one or more genes. We further explored the effects of the three significant SNPs (*CD44* rs187115, *IL-33* rs1929992, and *ST2* rs3821204) on their gene expression by investigating a public database, GTEx portal (https://gtexportal.org/) [24]. Statistical analysis of the data was performed using the statistical software package SPSS 24.0 (SPSS Inc., IBM, Chicago, IL, USA), and the test was performed using a two-sided test with a test level of *α* = 0.05. We used the false positive reporting probability (FPRP) to evaluate meaningful findings. We set 0.2 as the FPRP threshold, and 0.1 as the prior probability to detect the preponderance ratio (OR) associated with genotypes and haplotypes in the study to be 0.67/1.50 (protection/risk effect). Only significant results with an FPRP value of < 0.2 would be considered a noteworthy finding.

## 3. Results

### 3.1. Demographic and Clinical Characteristics of Participants

Initially, 593 subjects were enrolled in the HCC group, 21 cases had no pathological reports, and 7 cases had other cancers, and thus 28 cases were excluded. Ultimately, the study included 565 patients in the HCC group (males = 487, females = 78, age 10–89 years) and 561 in the healthy control group (males = 488, females = 73, age 22–78 years). Briefly, male participants showed a higher prevalence of HCC than females (487 vs. 78). Metastasis was found in 85 patients (15.0%). Stage of HCC was staged A or B in 259 patients (45.8%) and stage C or D in 306 patients (54.2%). Moreover, there were no statistical differences in the distributions of age, gender, smoking status, and alcohol consumption between the 2 groups. Detailed demographic and clinical characteristics of the study participants, including age, gender, smoking and drinking status, Barcelona clinic liver cancer (BCLC) stage, and metastasis status, are provided in Table 2.

### 3.2. Association between *CD44* rs187115 and Susceptibility and Clinicopathological Parameters of HCC

In this study, the distribution of *CD44* gene rs187115 in the HCC group (*p* = 0.58) and the control group (*p* = 0.74) was consistent with the HWE. The frequency distribution differences of the GG, GA, and AA genotypes in the 2 groups were statistically significant (*p* = 0.010). We used the AA genotype and A allele as a reference to analyze the risk of HCC. Logistic regression was used to adjust the impact of confounding factors such as gender and age and for calculating the OR value of rs187115 on the risk of HCC and 95% CI. Ultimately, it was found that compared with other genotype individuals, people carrying the homozygous GG genotype were 2.469 times more likely to develop HCC than those carrying the AA gene (*p* = 0.027, adjusted OR (OR_adj_) = 2.469, 95% CI: 1.110–5.492). Furthermore, individuals who carried the heterozygous AG genotype were 1.359 times more likely to develop HCC than individuals carrying the GG gene (*p* = 0.026, OR_adj_ = 1.359, 95% CI: 1.039–1.778). Moreover, compared with the A allele, individuals carrying the G allele had a 1.423 times higher risk of HCC (*p* = 0.003, OR = 1.423, 95% CI: 1.131–1.792), suggesting that the G allele mutation was associated with an increased risk of HCC. The *CD44* rs187115 polymorphism genotype and allele frequencies for the HCC and control groups are listed in Table 3. Also, we further studied the clinical status of *CD44* rs187115 gene polymorphism in HCC to assess whether there was a difference in the distribution of the rs187115 genotype among clinical subgroups. The results showed that the distribution of AG and GG genotypes of rs187115 in the hepatic fibrosis subgroup was significantly different (*p* = 0.038) (Table 4).

### 3.3. Association between *IL-33* rs1929992 and Susceptibility and Clinicopathological Parameters of HCC

The distribution of *IL-33*rs1929992 locus genotype in the HCC group (*p* = 0.084) and the control group (*p* = 0.750) was consistent with HWE, which indicates a good representation of the population. There was no significant difference in the frequency distribution of the GG, GA, and AA genotypes between the 2 groups (*p* = 0.597). Additionally, the results showed that rs1929992 genotyping of GA, G vector, and G allele was not associated with the risk of HCC (Table 3). Furthermore, there were no significant associations between *IL-33* rs1929992 and any of the clinicopathological parameters (Table 5).

### 3.4. Association between *ST2* rs3821204 and Susceptibility and Clinicopathological Parameters of HCC

The observed genotype frequency of *ST2* rs3821204 was consistent with the expected distribution of HWE in both the HCC group (*p* = 0.071) and the control group (*p* = 0.125). The frequency distribution of the GG, CG, and CC genotypes in the 2 groups was statistically significant (*p* ≤ 0.001). The risk of HCC development was analyzed using the AA genotype and the A allele as a reference. After adjusting for gender and age using logistic regression, we calculated the OR value and 95% CI of rs187115 locus for the risk of HCC. Finally, the results showed that individuals with rs3821204 CG+CC genotype were 1.647 times more at risk of developing HCC than those with the GG genotype (*p* ≤ 0.001, OR_adj_ = 1.647, 95% CI: 1.296–2.093) (Table 3). Similarly, individuals carrying the homozygous CC genotype were 2.208 times more likely to develop HCC than individuals carrying the GG gene (*p* ≤ 0.001, OR_adj_ = 2.208, 95% CI: 1.548–3.149). Furthermore, individuals who carried the heterozygous CG genotype were 1.483 times more likely to develop HCC than individuals carrying the GG gene (*p* = 0.003, OR_adj_ = 1.483, 95% CI: 1.147–1.916). Moreover, compared with the G allele, individuals carrying the C allele had a 1.532 times higher risk of HCC (*p* ≤ 0.001, OR = 1.527, 95% CI: 1.285–1.813), suggesting that the C allele mutation was associated with an increased risk of HCC. Also, we further studied the clinical status of *ST2* rs3821204 gene polymorphism in HCC; however, there was no significant difference in the distribution of the *ST2* rs3821204 genotype in the clinical subgroup analysis (*p* > 0.05) (Table 6).

### 3.5. Haplotype Frequencies

Results obtained from SHEsis software suggested that the 3 SNPs (*CD44*, *IL-33*, and *ST2*) were in a strong linkage disequilibrium. The risk of HCC was affected when cooccurrence of these polymorphisms was examined using logistic regression analysis. We found 3 haplotypes to be associated with the risk of HCC. Compared to participants with other haplotypes, carrying the rs187115-rs1929992-rs3821204 G-G-C haplotype increased the risk of HCC by 3.181 times (*p* ≤ 0.001, OR = 3.181, 95%CI = 1.664-6.082). The results of haplotype frequencies are summarized in Table 7, and Table 8 shows the FPRP values of significant results at different prior probability levels because their probability of being a false positive result was <20%.

### 3.6. Expression Quantitative Trait Loci

We further explored biological effects of the three significant SNPs (*CD44* rs187115, *IL-33* rs1929992, and *ST2* rs3821204) on their gene expression by investigating a public database (GTEx portal).We found that genotypes of these SNPs were not associated with their gene expression in liver tissue and whole blood cells.

## 4. Discussion

In this study, the results showed that the distribution frequency of the *CD44* rs187115 allele and genotype in the HCC case group and the control group was statistically significant (*p* < 0.05), which was consistent with the results of Liu et al. in lung cancer and Winder et al. in gastric adenocarcinoma [25, 26]. Also, we further showed that there was a significant difference in the distribution of the rs187115 and GG genotypes in the liver fibrosis subgroup. *CD44* belongs to the family of adhesion molecules and is also a hyaluronic acid receptor, which means it is mainly involved in the adhesion between cells and the interstitial matrix [27]. It was reported that *CD44* promoted HCC CSC stemness by regulating natural killer (NK) sensitivity or the tyrosine-protein kinase-Met-class I phosphoinositide 3-kinase-protein kinase B (c-Met-PI3K-AKT) signaling cascade, leading to poor prognoses of cancer and chemoresistance [28, 29]. Additionally, *CD44* is involved in the maintenance of CSCs in HCC, possibly through the PI3K/AKT/mTOR pathway and the NOTCH3 signaling pathway [30]. In some genes, an SNP located in a coding, promoter, or regulatory region, may have a certain function, whereas *CD44* SNP rs187115 is located in the first intron of *CD44* [31, 32]. Until this point, the regulatory mechanism of *CD44* intron 1 has not been reported, but it has been found that the polymorphism of the *CD44* rs187115 gene may act on chemical resistance and cellular stress response in a p53-dependent manner in HCC [33].

As a bifunctional factor, *IL-33* has both transcription factors and cytokine activity [34]. There is a strong correlation between the level of *IL-33* and the progression of disease in a variety of malignant epithelial tumors [19, 35]. Previous studies have shown that rs1929992 was associated with the risk of several autoimmune diseases. Since it is functional and has not reported its relationship to HCC, we studied whether it is associated with HCC risk [36–38]. However, the results showed that the difference in distribution frequency of rs1929992 locus alleles and genotypes in the HCC case group and control group was not statistically significant (*p* > 0.05), which was similar to the findings of Jafarzadeh et al. in breast cancer [39]. Some studies have reported overexpression of *IL-33* in colorectal cancer [19, 40], while in HCC, inconsistent results were observed. Zhang et al. found that increased *IL-33* protein levels were present in HCC patients' serum and liver tissue [41], whereas Bergis et al. did not find a significant difference in *IL-33* serum levels between HCC patients and healthy controls [42]. Thus, further replication studies with larger sample sizes are needed to identify the relationship of rs1929992 to the risk of HCC.

Recent studies have also found that soluble *ST2* levels were highly expressed in the liver, lung, and breast cancer; malignant glioma; and other tumor tissues; this was associated with the occurrence, invasion, and metastasis of tumors [43–45]. In the present study, we found that the frequency of the *ST2* rs3821204 C allele was higher in the HCC group than in the healthy control group; our results were consistent with the findings of Wei et al. on Chinese HCC patients [46]. The *ST2* rs3821204 is located in the three prime untranslated regions (3′UTR) of s*ST2* mRNA, and the CC genotype of rs3821204 is highly correlated with elevated plasma s*ST2* levels in vivo [47]. The *ST2* rs3821204 CC genotype may contribute to hepatocarcinogenesis by enhancing *ST2* production at the transcriptional and translational levels [46]. The physiological effect of *ST2* gene rs3821204 polymorphism on HCC patients is mainly exerted by enhancing the synthesis of *ST2* protein [46]. Additionally, studies have shown that *ST2* deficiency could prevent tumor progression in a mouse model [48]. Based on the above mechanisms, individuals with rs3821204 are prone to develop HCC.

In this study, by performing a haplotype analysis, we found that *CD44* rs187115, *IL-33* rs1929992, and *ST2* rs3821204 have a combined effect on HCC susceptibility. By analyzing the reasons, the development of HCC was related to various genetic polymorphisms, and changes in multiple genes could cause genetic and molecular abnormalities [49, 50]. There are several studies related to the *IL-33*/*ST2* axis and CSCs [51, 52]. Our previous research clarified that *IL-33* binds to its receptor *ST2* and induces phosphorylation of c-Jun N-terminal kinase activation (JNK), which leads to the expansion of colon cancer cell stemness [19]. Also, some researchers have found that *IL-33* can promote the activation of p38, increasing the levels of liver CSC markers expression, and epithelial to mesenchymal transition- (EMT-) like changes [53]. We suppose that *IL-33*/*ST2* may enhance *CD44* expression to initiate HCC, but further experimentation is required to elucidate the mechanism.

In summary, our genetic results suggest an association between SNPs (*CD44* rs187115, *ST2* rs3821204) and the risk of HCC. The combination of *CD44* rs187115, *IL-33* rs1929992, and *ST2* rs3821204 might be used as a marker to identify a subgroup at higher risk of HCC among the Chinese population. Unfortunately, the signaling pathway for HCC development from *CD44* to *IL-33*/*ST2* axis has not been reported, and the small sample size of this study may limit the applicability of these results. Therefore, future research will be required, recruiting larger sample sizes, careful design, and more clinical information to identify risk factors for HCC development from *CD44* rs187115, *IL-33* rs1929992, and *ST2* rs3821204 polymorphisms and the underlying biological mechanisms leading to HCC.

## Figures and Tables

**Table 1:** SNP oligonucleotide sequences used for related gene genotyping.

SNP	PCR primer
CD44 rs187115	Forward: 5′-TCAGGCAGGAGGAATAGGACA-3′ Reverse: 5′-CTCCTGCCCAATAAAGCCAA-3′
IL-33 rs1929992	Forward: 5′-TATGACACAGGACCCCGGAA -3′ Reverse: 5′- GAAGTCATCATCAACTTGGAACCT-3′
ST2 rs3821204	Forward: 5′-GACTGTTCCTGTTTGCTGGGA-3′ Reverse: 5′-TTGTTCACTTTACCACCCTCGC-3′

**Table 2:** General characteristics of HCC patients and the normal controls.

Characteristics	Cases (*n* = 565)	Controls (*n* = 561)	*p* value
Age (year)			0.546
Range	10-89	22-78	
Mean	53.62	52.15	
<40	95	102	
41-50	133	152	
51-60	186	176	
>60	151	131	
Gender			0.453
Male	487	488	
Female	78	73	
BMI (kg/m^2^)			0.406
<18.5	62	51	
18.5-23.9	366	359	
≥24	137	151	
Smoking status			0.734
No	344	336	
Yes	221	225	
Alcohol drinker			0.113
No	374	396	
Yes	191	165	
HBV infection			
HbsAg (−)	47	479	
HbsAg (+)	497	75	≤0.001
Liver cirrhosis			
Absent	487	491	
Present	78	70	0.51
BCLC stage			
A+B stage	259		
C+D stage	306		
Metastasis			
No	480		
Yes	85		

**Table 3:** Distribution and genotype frequencies of related polymorphisms in the HCC group and the control group.

Parameter	Case, *n* (%)	Controls, *n* (%)	OR (95% CI)	*p*_OR_	OR_adj_ (95% CI)	*p*_ORadj_
CD44 rs187115						
All						
AA	383 (67.8)	421 (75.0)	1.00		1.00	
AG	162 (28.7)	131 (23.4)	1.359 (1.039-1.778)	0.025	1.359 (1.039-1.778)	0.026
GG	20 (3.5)	9 (1.6)	2.443 (1.099-5.430)	0.028	2.469 (1.110-5.492)	0.027
AG+GG	182 (32.2)	140 (25.0)	1.429 (1.101-1.855)	0.007	1.429 (1.102-1.854)	0.007
Alleles						
A	928 (82.1)	973 (86.7)	1.00		1.00	
G	202 (17.9)	149 (13.3)	1.421 (1.129-1.789)	0.003	1.423 (1.131-1.792)	0.003
IL-33 rs1929992						
All						
AA	169 (29.9)	157 (28.0)	1.00		1.00	
GA	261 (46.2)	276 (49.2)	0.879 (0.667-1.157)	0.357	0.886 (0.672-1.168)	0.390
GG	135 (23.9)	128 (22.8)	0.980 (0.708-1.356)	0.902	0.980 (0.708-1.358)	0.906
AG+GG	396 (70.1)	404 (72.0)	0.911 (0.704-1.178)	0.476	0.916 (0.708-1.186)	0.506
Alleles						
A	599 (53.0)	590 (52.6)	1.00		1.00	
G	531 (47.0)	532 (47.4)	0.983 (0.833-1.160)	0.840	0.984 (0.834-1.161)	0.848
ST2 rs3821204						
All						
GG	198 (35.0)	264 (47.1)	1.00		1.00	
CG	255 (45.2)	230 (41.0)	1.478 (1.144-1.910)	0.003	1.483 (1.147-1.916)	0.003
CC	112 (19.8)	67 (11.9)	2.229 (1.564-3.177)	≤0.001	2.208 (1.548-3.149)	≤0.001
CG+CC	367 (65.0)	297 (52.9)	1.648 (1.297-2.093)	≤0.001	1.647 (1.296-2.093)	≤0.001
Alleles						
G	631 (56.0)	690 (61.5)	1.00		1.00	
C	499 (43.0)	432 (38.5)	1.532 (1.290-1.820)	≤0.001	1.527 (1.285-1.813)	≤0.001

OR: odds ratio; OR_adj_: adjusted odds ratio; CI: confidence interval.

**Table 4:** Association of CD44 rs187115 genotype with clinical characteristics in HCC patients.

Characteristics	rs187115			*p* value	rs187115		*p* value	rs187115		*p* value
	AA	AG	GG		AG	GG		AA	AG+GG	
Age (year)										
<40	147	57	6		57	6		147	63	
≥40	235	106	14	0.589	106	14	0.659	235	120	0.351
Gender										
Male	55	20	3		20	3		55	23	
Female	327	143	17	0.800	143	17	0.728	327	160	0.563
BMI (kg/m^2^)										
<18.5	43	16	4		16	4		43	20	
18.5-23.9	242	109	14		109	14		242	123	
≥24	97	38	2		38	2		97	40	
BCLC stage										
A+B stage	211	83	11		83	11		211	94	
C+D stage	171	80	9	0.634	80	9	0.730	171	89	0.373
Smoking status										
No	230	100	12		100	12		230	112	
Yes	152	63	8	0.973	63	8	0.907	152	71	0.840
Alcohol drinker										
No	251	107	15		107	15		251	122	
Yes	131	56	5	0.692	56	5	0.402	131	61	0.838
Metastasis										
No	272	181	17		181	17		272	198	
Yes	71	21	3	0.008	21	3	0.545	71	24	0.002
Liver cirrhosis										
Absent	116	53	2		53	2		116	55	
Present	266	110	18	0.117	110	18	0.038	266	128	0.955
HBV infection										
HbsAg (−)	37	18	1		18	1		37	19	
HbsAg (+)	339	141	18	0.675	141	18	0.419	339	159	0.754
HCV infection	6	4	1		4	1		6	5	
AST										
Negative	204	79	9		79	9		204	88	
Positive	178	84	11	0.460	84	11	0.770	178	95	0.226
ALT										
Negative	235	163	12		163	12		235	175	
Positive	147	67	8	≤0.001	67	8	0.309	147	75	0.031
GGT										
Negative	130	66	8		66	8		130	74	
Positive	252	97	12	0.322	97	12	0.966	252	109	0.132
AFP										
Negative	154	62	11		62	11		154	73	
Positive	228	101	9	0.343	101	9	0.144	228	120	0.580

ALT: alanine aminotransferase; AST: aspartate aminotransferase; GGT: *γ*-glutamyl transpeptidase; AFP: alpha fetoprotein.

**Table 5:** Association of IL-33 rs1929992 genotype with clinical characteristics in HCC patients.

Characteristics	rs1929992			*p* value	rs1929992		*p* value	rs1929992		*p* value
	AA	GA	GG		GA	GG		AA	GA+GG	
Age(year)										
Range	13-83	10-89	24-87		10-89	24-87		13-83	10-89	
Mean	52.6	52.1	54.3		52.1	54.3		52.6	53.1	
<40	23	51	15		51	15		23	663	
≥40	146	210	120	0.056	210	120	0.053	146	330	0.057
Gender										
Male	138	233	116		233	116		138	349	
Female	31	28	19	0.082	28	19	0.329	31	47	0.051
BMI (kg/m^2^)										
<18.5	15	32	14		32	14		15	46	
18.5-23.9	112	164	90		164	90		112	254	
≥24.0	42	65	31	0.816	65	31	0.735	42	96	0.182
BCLC stage										
A+B stage	90	145	71		145	71		90	216	
C+D stage	79	116	64	0.821	116	64	0.575	79	180	0.778
Smoking status										
No	98	166	80		166	80		98	246	
Yes	71	95	55	0.460	95	55	0.453	71	150	0.357
Alcohol drinker										
No	114	178	88		178	88		114	266	
Yes	55	83	47	0.831	83	47	0.398	55	130	0.948
Metastasis										
No	143	218	119		218	119		143	337	
Yes	26	43	16	0.470	43	16	0.221	26	59	0.882
Family history of cancer										
No	145	219	121		219	121		145	340	
Yes	24	42	14	0.302	42	14	0.121	24	56	0.985
Liver cirrhosis										
Absent	47	89	35		89	35		47	124	
Present	122	172	100	0.173	172	100	0.096	122	272	0.404
HBV infection										
HbsAg (−)	18	23	14		23	14		18	37	
HbsAg (+)	146	225	128		225	128		146	353	
HCV infection	5	3	3	0.684	3	3	0.792	5	6	0.379
AST										
Negative	82	135	76		135	76		82	211	
Positive	87	126	59	0.402	126	59	0.387	87	185	0.300
ALT										
Negative	97	155	94		155	94		97	249	
Positive	72	106	41	0.066	106	41	0.056	72	147	0.221
GGT										
Negative	65	106	56		106	56		65	162	
Positive	104	155	79	0.855	155	79	0.868	104	234	0.587
AFP										
Negative	67	108	53		108	53		67	161	
Positive	102	153	82	0.886	153	82	0.684	102	235	0.822

ALT, alanine aminotransferase; AST, aspartate aminotransferase; GGT, *γ*-glutamyl transpeptidase; AFP, alpha fetoprotein.

**Table 6:** Association of ST2 rs3821204 genotype with clinical characteristics in HCC patients.

Characteristics	rs3821204			*p* value	rs3821204		*p* value	rs3821204		*p* value
	GG	CG	CC		CG	CC		GG	CG+CC	
Age(year)										
Range	10-78	19-87	24-89		19-87	24-89		10-78	19-89	
Mean	52.8	52.2	52.9		52.2	52.9		52.8	52.6	
<40	28	36	15		36	15		28	51	
>40	170	220	97	0.981	220	97	0.864	170	317	0.926
Gender										
Male	176	219	92		219	92		176	311	
Female	22	36	20	0.250	36	20	0.359	22	56	0.173
BMI (kg/m^2^)										
<18.5	21	30	11		30	11		21	41	
18.5-23.9	125	168	74		168	74		125	242	
≥24.0	52	57	27	0.883	57	27	0.832	52	84	0.670
BCLC stage										
A+B stage	110	131	64		131	64		110	195	
C+D stage	88	124	48	0.501	124	48	0.308	88	172	0.582
Smoking status										
No	123	152	68		152	68		123	220	
Yes	75	103	44	0.863	103	44	0.842	75	147	0.613
Alcohol drinker										
No	131	165	77		165	77		131	242	
Yes	67	90	35	0.752	90	35	0.452	67	125	0.958
Metastasis										
No	122	211	95		211	95		122	306	
Yes	17	44	17	0.417	44	17	0.623	17	61	0.222
Family history of cancer										
No	167	221	96		221	96		167	317	
Yes	31	34	16	0.783	34	16	0.807	31	50	0.511
Liver cirrhosis										
Absent	62	77	31		77	31		62	108	
Present	136	178	81	0.798	178	81	0.626	136	259	0.641
HBV infection										
HbsAg (−)	16	11	11		11	11		16	22	
HbsAg (+)	179	239	99		239	99		179	338	
HCV infection	3	5	2	0.304	5	2	0.144	3	7	0.617
AST										
Negative	108	127	60		127	60		108	187	
Positive	90	128	52	0.575	128	52	0.506	90	180	0.415
ALT										
Negative	121	148	75		148	75		121	223	
Positive	77	107	37	0.271	107	37	0.107	77	144	0.936
GGT										
Negative	90	95	42		95	42		90	137	
Positive	108	160	70	0.821	160	70	0.964	108	230	0.060
AFP										
Negative	83	99	44		99	44		83	143	
Positive	115	156	68	0.789	156	68	0.933	115	224	0.494

ALT: alanine aminotransferase; AST: aspartate aminotransferase; GGT: *γ*-glutamyl transpeptidase; AFP: alpha fetoprotein.

**Table 7:** The association of haplotype of each gene with hepatocellular cancer risk.

Haplotype	Case (%) *N* = 565	Control (%) *N* = 561	*p*	OR (95% CI)
rs187115-rs1929992-rs3821204				
AAC	227.91 (0.202)	168.09 (0.150)	0.001	1.434 (1.152, 1.785)
AAG	264.26 (0.234)	343.55 (0.306)	≤0.001	0.692 (0.574, 0.834)
AGC	168.53 (0.149)	149.51 (0.133)	0.279	1.140 (0.899, 1.446)
AGG	267.30 (0.237)	310.86 (0.277)	0.027	0.808 (0.669, 0.977)
GAC	44.16 (0.039)	34.13 (0.030)	0.261	1.296 (0.823, 2.042)
GAG	62.66 (0.055)	44.23 (0.039)	0.073	1.431 (0.965, 2.122)
GGC	38.40 (0.034)	12.27 (0.011)	≤0.001	3.181 (1.664, 6.082)
GGG	56.77 (0.050)	59.36 (0.053)	0.774	0.947 (0.652, 1.376)
rs187115, rs3821204				
AC	396.22 (0.351)	318.60 (0.284)	≤0.001	1.362 (1.139, 1.627)
AG	531.77 (0.471)	653.40 (0.582)	≤0.001	0.638 (0.540, 0.753)
GC	82.77 (0.073)	45.40 (0.040)	≤0.001	1.874 (1.292, 2.719)
GG	119.23 (0.106)	104.60 (0.093)	0.330	1.147 (0.870, 1.513)
rs187115, rs1929992				
AA	492.04 (0.435)	511.85 (0.456)	0.321	0.919 (0.779, 1.086)
AG	435.96 (0.386)	460.15 (0.410)	0.238	0.903 (0.763, 1.070)
GA	106.96 (0.095)	78.15 (0.070)	0.031	1.396 (1.030, 1.893)
GG	95.04 (0.084	71.85 (0.064)	0.069	1.342 (0.976, 1.845)
rs1929992, rs3821204				
AC	271.98 (0.241)	200.95 (0.179)	0.003	1.453 (1.184, 1.783)
AG	327.02 (0.289)	389.05 (0.347)	0.003	0.767 (0.642, 0.917)
GC	207.02 (0.183)	163.05 (0.145)	0.015	1.319 (1.054, 1.651)
GG	323.98 (0.287)	368.95 (0.329)	0.030	0.820 (0.686, 0.981)

**Table 8:** False positive report probability values for associations between the risk of HCC and the frequency of genotypes and haplotypes of the CD44 gene and ST2 dene in an Chinese population.

Genotype/haplotype	OR (95% CI)	*p* value	Statistical power^a^	Prior probability				
				0.25	0.1	0.01	0.001	0.0001
CD44 rs187115								
AG/AA	1.359 (1.039-1.778)	0.025	0.764	0.091	0.232	0.796	0.971	0.997
GG/AA	2.443 (1.099-5.430)	0.028	0.116	0.429	0.693	0.961	0.996	1.000
AG+GG/AA	1.429 (1.101-1.855)	0.007	0.642	0.034	0.095	0.563	0.921	0.991
ST2 rs3821204								
CG/GG	1.478 (1.144-1.910)	0.003	0.545	0.016	0.046	0.345	0.842	0.982
CC/GG	2.229 (1.564-3.177)	≤0.001	0.014	0.002	0.006	0.065	0.411	0.875
CG+CC/GG	1.648 (1.297-2.093)	≤0.001	0.220	0.001	0.002	0.020	0.168	0.668

^a^Statistical power was calculated using the number of observations in the subgroup and the OR and *p* values in this table.

## Data Availability

The datasets used and analyzed during the current study are available from the corresponding author on reasonable request.
